# Preoperative evaluation of endoscopic thyroidectomy via the total areola approach (ETA): a fluid-structure interaction model for predicting lymph node clearance and surgical suitability

**DOI:** 10.3389/fbioe.2025.1599770

**Published:** 2025-08-18

**Authors:** Ping Li, Yuheng Du, Xudong Wang, Yu Shi, Chongyang Ye, Rui Jin

**Affiliations:** ^1^ Key Laboratory of Basic an Translational Medicine on Head and Neck Cancer, Department of Maxillofacial and Ear, Nose and Throat Oncology, Tianjin Medical University Cancer Institute and Hospital, National Clinical Research Center for Cancer, Tianjin, China; ^2^ College of Arts, South China Agricultural University, Guangzhou, Guangdong, China; ^3^ School of Fashion and Textiles, The Hong Kong Polytechnic University, Kowloon, Hong Kong SAR, China

**Keywords:** technology of total areola approach, fluid-solid interaction model, central lymph node dissection, tissue deformation, innominate artery velocity

## Abstract

The global increase in thyroid cancer incidence has driven the adoption of minimally invasive techniques, such as endoscopic thyroidectomy via the total areola approach (ETA), which is widely used in China. However, concerns persist regarding the completeness of central lymph node dissection (CLND) in ETA due to anatomical constraints (e.g., clavicle and sternum), which may obscure the surgical view of the upper diaphragm (level VII, defined as the region between the clavicular surface and innominate artery). Clinical reports of residual/recurrent lymph nodes in ETA patients underscore the need for precise preoperative evaluation. We retrospectively analyzed 513 patients with T1–T2 thyroid cancer (178 ETA, 335 open surgery) who underwent CLND. Preoperative CT imaging was used to construct a fluid-solid interaction model simulating tissue deformation and stress under 0.5–2 N traction forces, with innominate artery flow velocities predicted computationally. Patients were stratified by clavicle-to-innominate artery distance: <5 mm, 5–13 mm, and >13 mm. No significant difference in lymph node yield was observed between the <5 mm and 5–13 mm groups compared to open surgery. However, the >13 mm group had significantly fewer dissected nodes (p < 0.05), with three recurrence cases during follow-up. ETA achieves oncologic outcomes comparable to open surgery for patients with clavicle-to-innominate artery distances <13 mm. Beyond this threshold, incomplete dissection may occur, suggesting preoperative CT assessment of this anatomical parameter could guide surgical approach selection.

## 1 Introduction

Thyroid cancer accounted for 586,000 cases worldwide in 2020, ranking as the 9th most common cancer by incidence (Sung et al., 2021). Papillary thyroid carcinoma (PTC), the most prevalent endocrine malignancy, has shown a continuous increase in global incidence over the past 2 decades (La Vecchia et al., 2015). Surgery remains the primary treatment for thyroid cancer; however, conventional open thyroidectomy (COT) often leaves visible neck scars, which are particularly undesirable for patients with high cosmetic expectations, such as young women and Asian populations, who are prone to pronounced scarring (Wang et al., 2015). To address this, scar-free surgical approaches have been developed, with the transoral endoscopic thyroidectomy vestibular approach (TOETVA) and total endoscopic thyroidectomy via the areola approach (ETA) being the most widely adopted. These techniques have gained popularity in PTC treatment as surgical proficiency has improved (Yang et al., 2015; Liu et al., 2021; Anuwong et al., 2018).

In China, ETA is particularly favored due to its alignment with national surgical traditions and its intrinsic advantages, such as facilitating the dissection of the superior gland pole, the berry ligament, and the external branch of the superior laryngeal nerve. This approach also enables effective clearance of level II lymph nodes and achieves excellent cosmetic outcomes (Liang et al., 2015; Zhang et al., 2019; Jia et al., 2017). However, a notable limitation of ETA is the potential difficulty in dissecting the most caudal portion of the central compartment lymph nodes due to endoscopic visual obstruction and instrument interference from the clavicles and sternum. This challenge may contribute to central neck recurrence observed in some patients post-ETA, though the exact relationship remains unclear. Additionally, revision surgery for recurrence carries risks of complications, including hoarseness and damage to the trachea or esophagus.

The level VII lymph node compartment, as defined in the eighth edition of the AJCC Cancer Staging Manual, is bounded superiorly by the suprasternal notch, inferiorly by the innominate artery, anteriorly by the sternum, and posteriorly by the trachea, esophagus, and prevertebral fascia. Lymph nodes that are too numerous or poorly visualized on CT scans are excluded from study analysis. Importantly, soft tissue dissection is also critical, as PTC, particularly the infiltrative type, frequently metastasizes to these regions.

From our experience and that of other centers, COT provides clear visualization of the innominate artery. However, the traction force applied during surgery can cause tissue deformation, and the depth of level VII varies among patients, making it unclear which cases are suitable for complete clearance. Furthermore, there is limited research on the rationale for dissecting levels VI and VII lymph nodes from a caudal-to-cranial approach. In our hospital, some ETA patients have experienced recurrence in level VII lymph nodes or soft tissue, followed by neck metastasis during follow-up. Reviewing surgical videos, we noted that the innominate artery was neither visible nor palpable in these cases, suggesting that patients with a relatively lower innominate artery may not achieve complete clearance of level VII tissues. Therefore, identifying PTC patients unsuitable for ETA is of significant clinical importance.

To address these issues, we aim to evaluate whether the traction force applied during surgery effectively removes target tissues. Proper traction enhances visualization of the thyroid gland, surrounding structures, and lymph nodes, facilitating precise dissection while minimizing injury to critical structures such as the recurrent laryngeal nerve and parathyroid glands. However, excessive traction can cause tissue trauma, bleeding, or incomplete dissection, whereas insufficient traction may lead to inadequate exposure and residual disease. Thus, understanding the relationship between traction force and tissue deformation is essential. Additionally, traction-induced tissue deformation may alter nearby vascular structures, including the innominate artery, necessitating an assessment of its hemodynamic responses.

Previous studies have utilized the finite element (FE) method to simulate biomechanical responses in both *in vitro* and *in vivo* settings, including tissue stress, pressure, and deformation (Li et al., 2021; Shi et al., 2024; Serpell et al., 2015). For instance, Serpell et al. applied numerical simulation method to predict differential levels of tension within each RLN during ETA, which indicated that The stiffer left RLN and the higher tension generated in the right RLN during thyroidectomy may jointly contribute to the higher right RLN palsy rat (Serpell et al., 2015), while Bauman et al. simulated the entire thorax system, including ribs, cartilages, sternum, vertebrae, intervertebral discs, muscles, and major organs, to analyze stress and deformation (Bauman et al., 2022). Building on these approaches, we will apply the FE method to predict tissue deformation and hemodynamic changes in the innominate artery.

In this study, we systematically collected data from ETA patients, including CT scans and recurrence data, combined with fine-needle aspiration (FNA) analysis and CT imaging to monitor the endoscopic surgical process. Based on tissue depth, patients were classified into three groups: <5 mm, 5–13 mm, and 13–25 mm. To further investigate biomechanical responses during ETA, we constructed a fluid-solid interaction (FSI) model to numerically simulate tissue deformation and innominate artery hemodynamics under traction forces of 0.5 N, 1 N, and 2 N. These simulations aim to optimize surgical techniques, minimize residual disease, and establish criteria for determining the suitability of PTC patients for ETA.

## 2 Methodology

### 2.1 Study design and perioperative preparation

A retrospective study was conducted on eligible patients who underwent unilateral lobectomy and central neck dissection, as recommended by the American Thyroid Association (ATA) and Chinese Thyroid Management Guidelines (Haugen et al., 2016; Association, 2012), at the Department of Maxillofacial and Ear, Nose, and Throat Oncology, Tianjin Medical University Cancer Institute and Hospital (TJMUCH), between July 2018 and May 2020. The study was approved by the Institutional Review Board of TJMUCH (approval number: bc20241340).

The inclusion criteria for patients undergoing endoscopic thyroidectomy via the ETA were as follows: for benign tumors, the maximum nodule diameter ranged from 4 to 9 cm; for malignant tumors, the maximum nodule diameter was less than 2 cm, with no suspicious lateral neck metastatic lymph nodes detected on preoperative ultrasound or CT imaging. Patients with T4 lesions involving nerves or the trachea, infectious thyroid diseases, or parathyroid diseases were excluded. The resection scope followed the American Joint Committee on Cancer (AJCC) 2017 guidelines and Chinese guidelines. For malignant cases, the procedure included at least unilateral thyroidectomy with isthmus resection and ipsilateral central lymph node dissection, or total thyroidectomy with bilateral central lymph node dissection.

In this study, 225 patients underwent ETA. A 1 cm observation port was created at the medial edge of the right areola for endoscope placement, and two 0.5 cm operative ports were established at the lateral edges of the bilateral areola for trocar placement. The surgical procedure involved creating a working space, opening the cervical midline, exposing the thyroid gland, dissecting critical structures such as the recurrent laryngeal nerve and parathyroid glands, and removing the affected thyroid tissue. Unilateral or bilateral central lymph node dissection was performed concurrently when indicated.

Exclusion criteria included benign tumors without central lymph node dissection (45 cases) and special types of thyroid cancer, such as medullary carcinoma and undifferentiated carcinoma. Ultimately, 178 patients who underwent either unilateral thyroidectomy with ipsilateral central lymph node dissection or total thyroidectomy with bilateral central lymph node dissection were included in this retrospective study. All patients were followed up until January 2023. Intraoperative recurrent laryngeal nerve monitoring was performed in all cases. Prior to surgery, patients were thoroughly informed about the three surgical approaches and allowed to freely choose their preferred method, providing written informed consent.

Data were collected from patient medical records, and a database was established to document the surgical approach, patient demographics (sex and age), tumor characteristics (number of lesions and diameter of the largest tumor), and the presence of Hashimoto’s thyroiditis. Central lymph node dissection (CLND)-related data included the total number of central lymph nodes and the number of metastatic central lymph nodes. Follow-up data included central compartment neck dissection (CCND) recurrence, as summarized in Table 2.

### 2.2 Geometry model of soft tissue and innominate artery

To determine the biomechanical responses of the soft tissue and innominate artery, we selected one of patient CT slices for analyzing the tissue deformation, tissue stress, and innominate artery hemodynamics by the traction force, which help doctors to understand the biomechanical mechanisms to identify PTC patients unsuitable for ETA is of significant clinical importance.

The geometric model was reconstructed based on computed tomography (CT) images. Using the extracted CT slices as masks, the three-dimensional (3D) geometry of the soft tissue and artery was reconstructed using Mimics software v20.0 (Materialise, Hungary). The reconstructed model was then imported into Ansys SpaceClaim software, where reverse engineering techniques were applied to generate a 3D solid model of the soft tissue and artery. The soft tissue thickness was defined as three groups to represent varying anatomical conditions. Finally, the 3D geometric model of the soft tissue and innominate artery was imported into Ansys Workbench v19.2 (ANSYS, Pennsylvania, Pittsburgh, United States) for numerical simulation of tissue deformation, stress distribution, and hemodynamic responses in the innominate artery.

### 2.3 Analysis of innominate artery flow

The Navier-Stokes (N-S) equations and the fluid continuity (F-C) equation were applied to predict the hemodynamic responses of the blood system (Spurk et al., 2020). These equations are expressed as follows (Equation 1):

$$\frac{\partial v}{\partial t}+\left(v\cdot \nabla \right)v=f-\frac{1}{p}\nabla p+\mu \nabla^{2}v\nabla v=0$$ (1)

Where *v* and *P* are the innominate artery flow velocity and the innominate artery flow hydrostatic pressure, and *μ* and *ρ* are the innominate artery flow viscosity coefficient and innominate artery density, respectively. The N-S equations are applied to characterize the relationship between the innominate artery hydrostatic pressure and innominate artery flow velocities, and the fluid continuity equation can be applied to detect the relationship between the innominate artery wall deformation and blood flow.

The Reynolds number (*Re*) is an important dimensionless quantity in fluid mechanics that is applied to determine the flow patterns in the different fluid flow situations. It can be expressed as below (Equation 2),

$$Re=\frac{pvL}{\mu }$$ (2)

Where *L* is the length of the innominate artery flow. The flow of the innominate artery flow could be regarded as laminar flow with the *Re* values less than 2,300. Which indicated that the direction of the innominate artery flow parallels to the innominate artery wall. Without the traction force on the soft tissue, the innominate artery wall can be regarded as laminar flow. And the blood flow density is approximate 1060 kg/m3 (Moser and Kenner, 1988), and the viscosity coefficient of the blood flow is nearly 0.003 Pa × s (Sun G. et al., 2020).

### 2.4 FSI model of tissue-artery system

The soft tissue can be assumed as a New-Hookean non-linear hyperelastic model, which can be expressed as follow (Equation 3)

$$W=C_{1}\left({Ī}_{1}-3\right)+\frac{1}{D_{1}}{\left(J-3\right)}^{2}$$ (3)

where *W* denotes the strain energy density, and the parameters *C*_1_ and *D*_1_ are approximately 5,000 Pa and 1.4 × 10–^7^ Pa^−1^, respectively (Ye et al., 2023). *I*_1_ is the first invariant of the Cauchy-Green deformation tensor and *J* is the volume ratio. *C*_1_ and *D*_1_ can be expressed as below under a linear elastic condition (Equation 4),

$$C_{1}=\frac{\mu }{2};D_{1}=\frac{\lambda }{2}$$ (4)

where *μ* and *λ* are shear modulus and bulk modulus, respectively.

The artery wall can be assumed as linear elastic model, where the Young’s modulus and Poisson’s ratio of the artery wall are 5 MPa and 0.49 (Holzapfel and Weizsäcker, 1998), respectively. The soft tissue and artery model was meshed by using linear tetrahedron elements with the 1 mm element size. The elements and nodes number are approximately 30,000 and 70,000, respectively. The mesh quality was about 0.76, indicating good mesh quality applied in our developed FE tissue-artery model.

**FIGURE 1:**
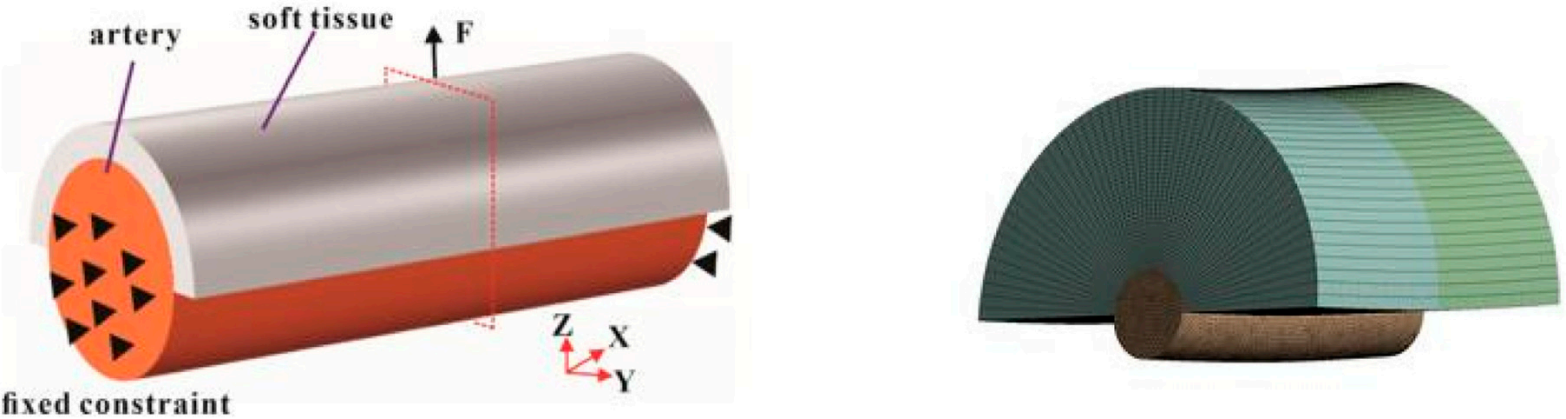
The force analysis and mesh of the tissue-artery model.

For the boundary conditions, the traction force (0.5 N, 1 N, and 2 N) was applied to simulate the clamping force during surgery (Figure 1). Both ends of the arterial tissue are set as fixed supported to provide the constraint for the FE model. The numbers of steps was set as 1 s, and the step end time was set as steady state. The simulated tissue deformation and stress was applied to assess whether lymph nodes and soft tissues in Zone 7 can be completely cleared *in vitro*.

**FIGURE 2:**
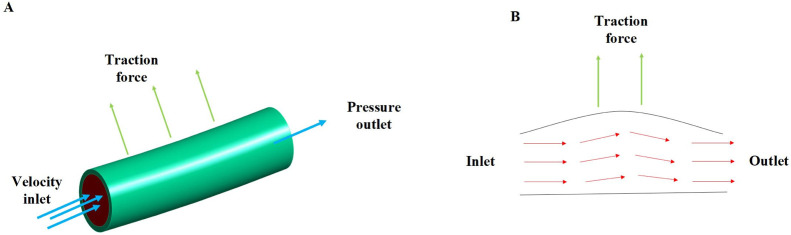
The innominate artery flow analysis; **(A)** the boundary conditions of innominate artery flow; **(B)** the innominate artery flow biomechanical mechanisms under the traction force.

The constructed 3D geometric innominate artery models and determined mechanical properties of innominate artery were input into the Ansys workbench Fluent for analyzing the artery hemodynamics influenced by the traction force in a 3D scale by using FE and finite volume (FV) method. The SIMPLE solution would be applied to analyze the innominate artery hemodynamics response of the traction force, which has good stability and convergence. The innominate artery models were meshed using the fluent Hexahedron elements with three boundary layers as well as 1 mm meshing size. For the boundary conditions, the velocity inlet and pressure outlet was applied in our proposed FSI model. Among them, the average velocity inlet for the innominate artery was approximately 60 cm/s and the 0 Pa pressure outlet was applied in this model, as the innominate artery was free outflow (Figure 2). The dynamic mesh was applied in the FSI model at the FSI interaction wall through smoothing, layering and re-meshing methods. The time step of the FSI coupling system was set as 20 and the iteration number of fluent was set as 1000, respectively.

### 2.5 Validation of our developed FE model of tissue-artery

**FIGURE 3:**
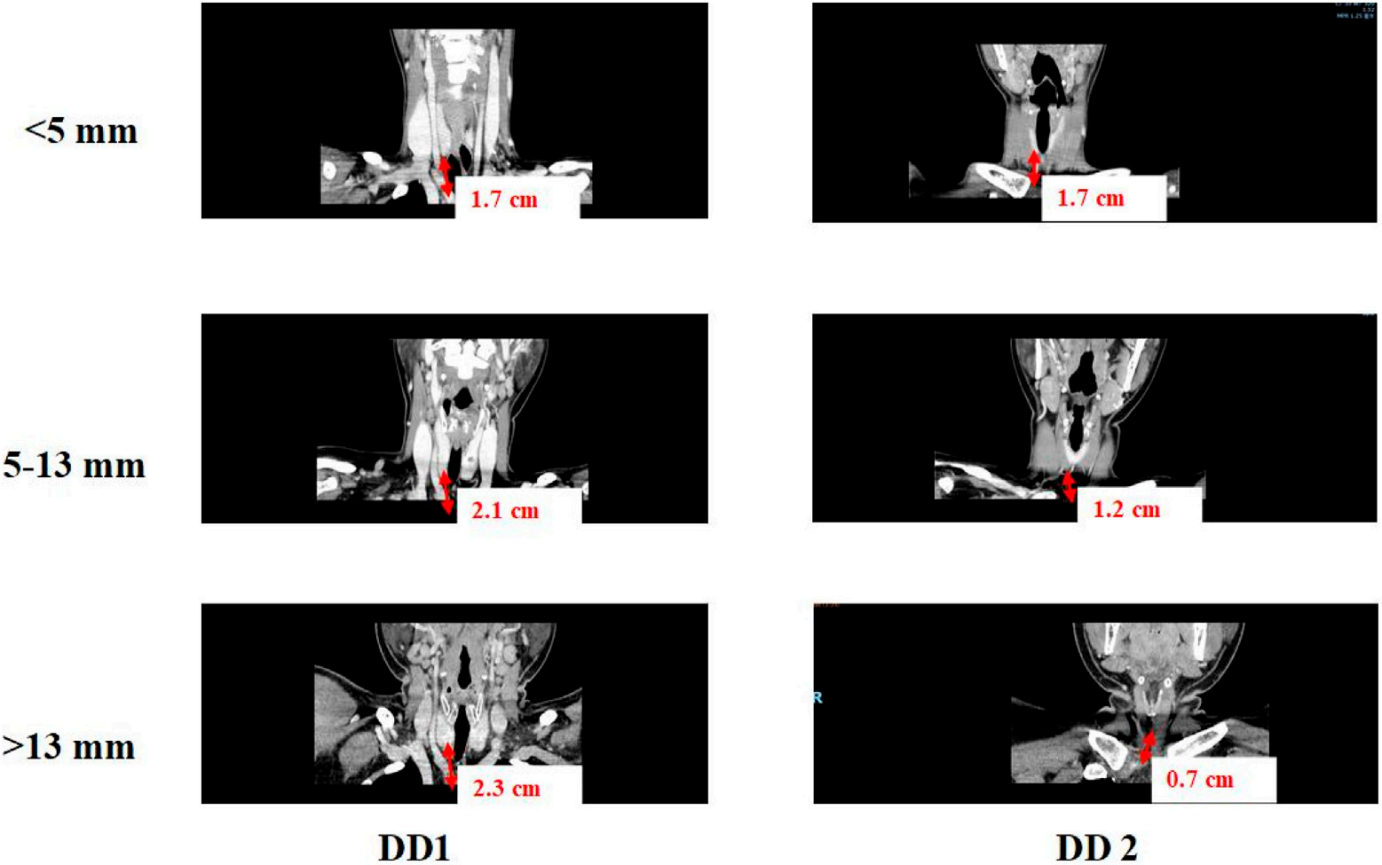
CT images to determine the values of DD1 and DD2.

CT scanning data was applied to validate the tissue deformation predicted by FSI model of tissue-artery system (Figure 3). Base on the CT scanning images, DD1 was defined as the distance from the inferior pole of the thyroid gland to the innominate artery, and DD2 was defined as the distance from the lower pole of thyroid gland to the clavicular head. The DD3 was defined as the distance from the clavicular head to the innominate artery. The clinic data were analyzed by one-way ANOVA, Welch ANOVA, and the χ^2^ test using Origin software (version 9.0; OriginLab Corporation). Differences with *P* values <0.05 were considered statistically significant. If the tissue deformation was more than DD3, which indicated that our lymph nodes and soft tissues in Zone 7 were fully cleared.

## 3 Results and discussion

### 3.1 Baseline characteristics for ETA in different groups

CT scan data from 178 patients who underwent endoscopic thyroidectomy via the ETA were analyzed in this section. The value of DD1 (the distance from the inferior pole of the thyroid gland to the innominate artery) ranged from 0.38 mm to 4.4 cm, with a median of 1.9 cm and a mean of 1.95 ± 0.7 cm. The value of DD2 (the distance from the inferior pole of the thyroid gland to the clavicular head) ranged from 0.05 cm to 3.7 cm, with a median of 1.37 cm and a mean of 1.42 ± 0.8 cm. Correspondingly, DD3 (the distance from the clavicular head to the innominate artery) ranged from −1.2 cm to 2.1 cm, with a median of 0.54 cm and a mean of 0.52 ± 0.7 cm.

For patients with DD3 less than 0, the central soft tissue and lymph nodes were visible within the surgical field. However, for patients with DD3 greater than 0, the central lymph nodes were hidden behind the clavicular bone and sternum, making them invisible from a bottom-to-top perspective during ETA. In such cases, thorough dissection of this tissue requires traction, which is critical for achieving complete clearance.

Based on the clinical data, soft tissue with a thickness of 5–13 mm was found to be more easily mobilized under physiological traction. Complete clearance is achieved within 5 mm, with no visual blind spots. For lesions between 5 and 13 mm, thorough clearance can generally be attained through the surgeon’s expertise and meticulous technique. Beyond 13 mm, visual blind spots may occur, increasing the risk of residual central lymph nodes. Additionally, Using 5 mm and 13 mm as grouping thresholds, the patients were divided into three groups: <5 mm, 5–13 mm, and >13 mm, with a maximum distance of 20 mm for group 3 (Table 1). The differences in the number of lymph nodes dissected and recurrence rates among the three groups were compared. According to the clinical data and tissue thickness groups, the average values of DD3 were 0.01 cm, 0.86 cm, and 1.63 cm for the <5 mm, 5–13 mm, and >13 mm groups, respectively. These findings highlight the importance of preoperative assessment of tissue thickness and anatomical relationships to determine the feasibility of ETA and ensure complete lymph node dissection.

**TABLE 1:** Baseline characteristics of the three groups.

	Whole	low (<5mm)	Middle (5-13mm)	Large (>13mm)
Age (years), mean±SD (range)	36.01±8.78	35.34±8.87	36.09±8.96	38.13±7.83
Sex				
Male	19	7	9	3
female	159(89.33%)	78(91.76%)	60(86.96%	21(87.5%)
Number of lesion				
Signal	128	62	49	17
Multiple	50	23	20	7
Extent of thyroidectomy and extent of CND				
Lobectomy &Unilateral CND	160	76	63	21
Total thyroidectomy &bilateral CND	18	9	6	3
Diameter of largest tumor (cm)	0.84 ± 0.43	0.89 ± 0.39	0.73 ± 0.41	0.88 ± 0.52
Hashimoto’s thyroiditis				
Yes	107	52	42	13
No	71	33	27	11

### 3.2 Determination tissue deformation by traction force

**FIGURE 4:**
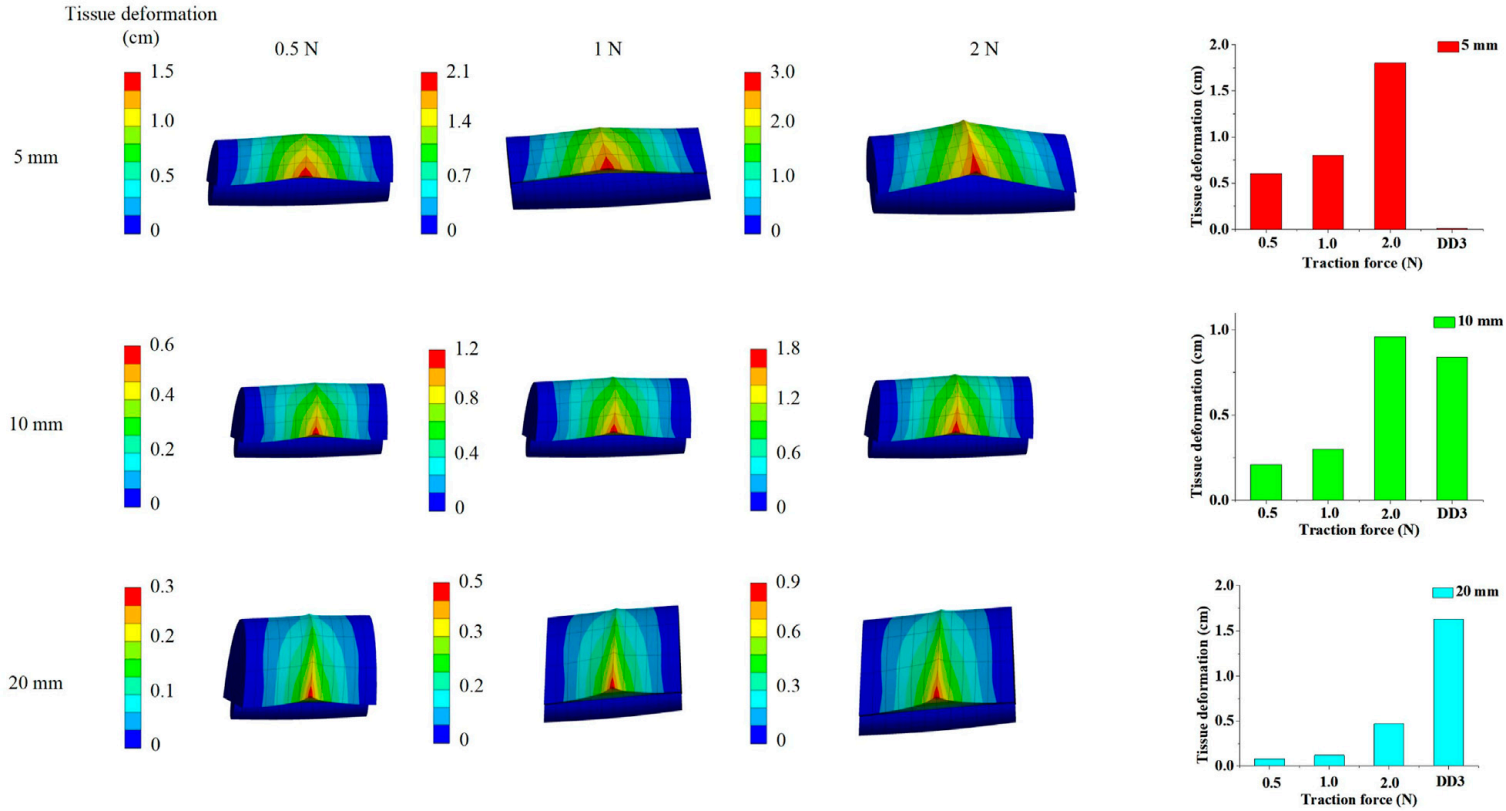
Numerical simulation results to determine tissue deformation by traction force.

Based on the results from Section 3.1, the tissue deformation for 5 mm, 10 mm, and 20 mm tissue thickness groups under traction forces ranging from 0.5 to 2 N was estimated using the FSI model. As shown in Figure 4, under a traction force of 1 N, the average tissue deformation was 0.8 cm, 0.3 cm, and 0.12 cm for tissue thicknesses of 5 mm, 10 mm, and 20 mm, respectively. When the traction force was increased to 2 N, the tissue deformation increased to 1.8 cm, 0.96 cm, and 0.47 cm for the corresponding tissue thicknesses of 5 mm, 10 mm, and 20 mm, respectively. These results demonstrate that higher traction forces lead to greater tissue deformation, while thicker tissue results in less deformation under the same traction force.

The simulated tissue deformation for the <5 mm group under traction forces of 0.5 N, 1 N, and 2 N exceeded 0.01 cm, and the deformation for the 5–13 mm group under a 2 N traction force exceeded 0.84 cm. However, the simulated tissue deformation for the >13 mm group remained below 1.63 cm under all tested traction forces. This indicates that lymph nodes and soft tissues in Zone 7 were completely cleared with traction forces of 0.5–2 N for patients with soft tissue thicknesses of <13 mm. In contrast, for patients with soft tissue thicknesses >13 mm, the lymph nodes and soft tissues in Zone 7 were not fully cleared, even with traction forces up to 2 N.

These findings suggest that ETA is effective for achieving complete clearance of Zone 7 lymph nodes and soft tissues in patients with soft tissue thicknesses <13 mm, while alternative surgical approaches should be considered for patients with thicker soft tissue (>13 mm) to ensure thorough dissection and minimize the risk of residual disease.

### 3.3 Determination tissue stress by traction force

**FIGURE 5:**
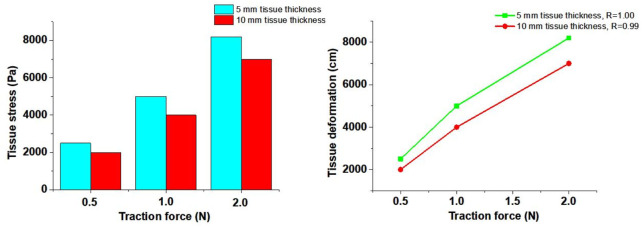
Numerical simulation results to determine tissue stress by traction force.

In this study, the 5 mm and 10 mm tissue thickness groups were used to simulate the tissue stress on the artery wall under traction forces. As illustrated in Figure 5, stress concentration was observed in the soft tissue during traction loading. The stress near the loading position was significantly higher than that at distant positions, and stress concentration also occurred at the edges of the soft tissue. The stress on the soft tissue increased with higher traction forces but decreased with greater soft tissue thickness.

The tissue stress on the innominate artery wall was approximately 2000 Pa, 4,000 Pa, and 7,000 Pa under traction forces of 0.5 N, 1 N, and 2 N, respectively, for the 10 mm tissue thickness group. Similarly, for the 5 mm tissue thickness group, the tissue stress on the innominate artery wall was approximately 2,500 Pa, 5,000 Pa, and 8,200 Pa under traction forces of 0.5 N, 1 N, and 2 N, respectively. The Pearson Correlation Analysis showed that a significant positive correlation was observed between tissue stress and traction force (r = 1.00 with 5 mm tissue thickness and r = 0.99 with 10 mm tissue thickness, p < 0.05). A linear relationship between traction force and tissue stress was observed at forces below 1 N. However, when the traction force reached 2 N, the tissue exhibited large deformation and nonlinear mechanical behavior. These results indicate that increased tissue stress can lead to expansion of the innominate artery wall, potentially influencing hemodynamic responses.

### 3.4 Determination artery hemodynamics responses by traction force

The N-S and F-C equations demonstrated that the innominate artery flow velocities are influenced by the traction force applied to the soft tissue. This traction force causes deformation of the artery wall, altering the innominate artery flow direction so that it is no longer parallel to its original orientation. This change in flow direction leads to an increase in innominate artery flow velocities and blood pressure along the direction of flow.

**FIGURE 6:**
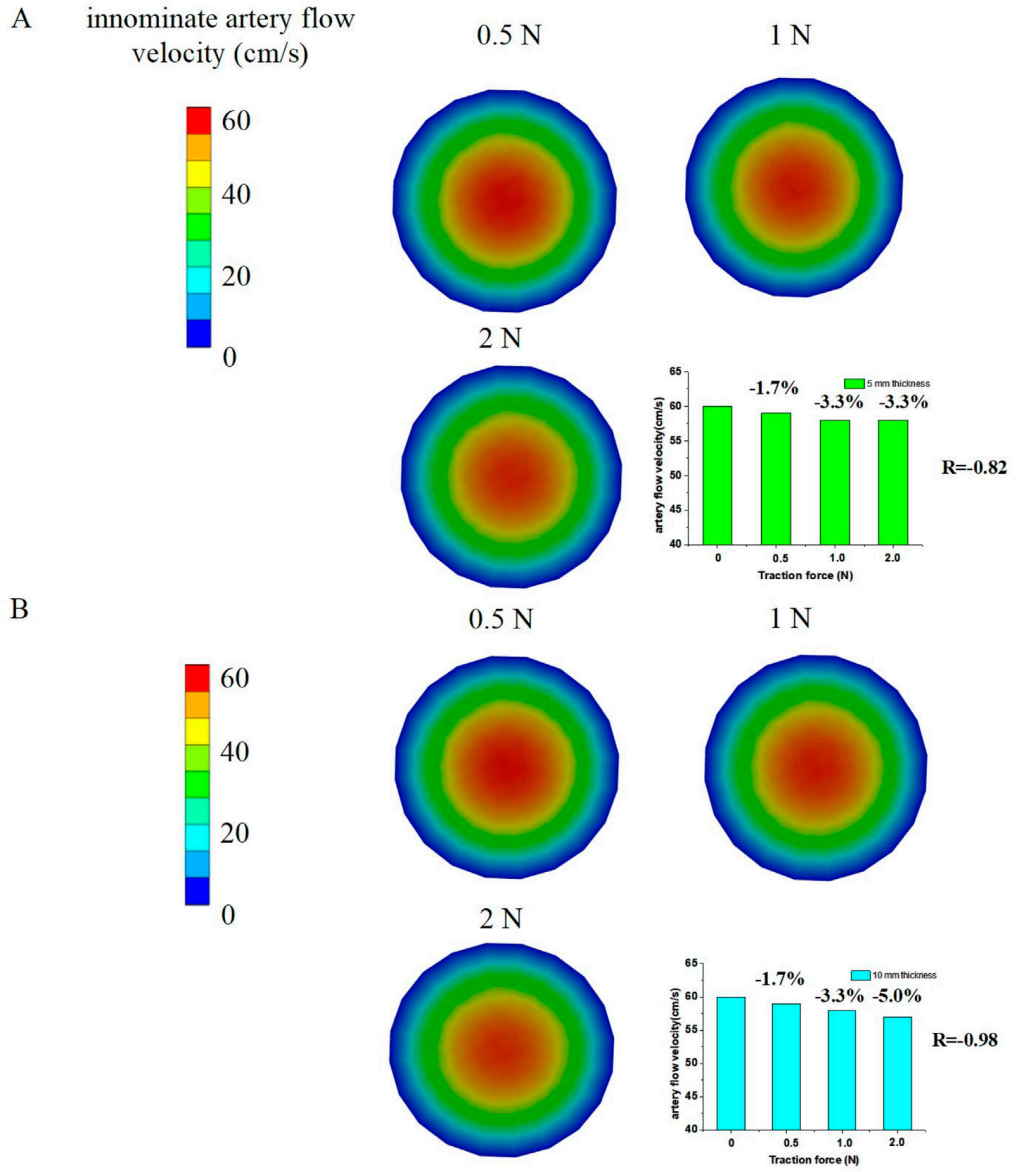
Numerical simulation results to determine innominate artery flow velocities by traction force; **(A)** 5 mm tissue thickness; **(B)** 10 mm tissue thickness.

The 3D FSI models revealed a decrease in innominate artery flow velocities, as illustrated in Figure 6. The innominate artery flow velocity mappings were simulated using FSI numerical models. The results showed that the innominate artery flow velocities decreased to approximately 59 cm/s (−1.7%), 58 cm/s (−3.3%), and 58 cm/s (−3.3%) under traction forces of 0.5 N, 1 N, and 2 N, respectively, for the 5 mm tissue thickness group. Similarly, for the 10 mm tissue thickness group, the flow velocities decreased to approximately 59 cm/s (−1.7%), 58 cm/s (−3.3%), and 57 cm/s (−5.0%) under the same traction forces. The Pearson Correlation Analysis showed that a significant negative correlation was observed between innominate artery flow velocity and traction force (r = −0.87 with 5 mm tissue thickness and r = −0.98 with 10 mm tissue thickness, p < 0.05). These findings indicate that the innominate artery flow velocity is reduced by the application of traction force.

Additionally, the innominate artery velocity values decreased from the core (venous center) to the margin (innominate artery wall) of the vein cross-sections. The simulated innominate artery velocities gradually decreased from the center to the venous wall in the 3D FSI models, a phenomenon attributed to the viscosity of the arterial flow.

These results highlight the impact of traction force on hemodynamics during endoscopic thyroidectomy via the ETA. The deformation of the artery wall and subsequent changes in flow velocity and direction underscore the need for careful intraoperative management to minimize hemodynamic disturbances and ensure patient safety.

### 3.5 Determination CNN node by traction force

Based on clinical data and simulated tissue deformation, when the maximum traction force applied was 0.5 N, 1 N, and 2 N, the central compartment neck dissection (CCND) could only fully remove lymph nodes in the <5 mm group. When the maximum traction force reached 2 N, the results suggested that CCND could also fully remove lymph nodes in the 5–13 mm group. However, for distances greater than 20 mm, even a maximum traction force of 2 N may not be sufficient to completely remove lymph nodes from a bottom-to-top approach, which was consistent with the simulation results. Analysis of the clavicle-to-innominate artery distance revealed a critical threshold at 13 mm. While patients with distances <13 mm (including both <5 mm and 5–13 mm subgroups) showed comparable lymph node yields to open surgery (p > 0.05), those with distances >13 mm demonstrated significantly reduced nodal retrieval (p < 0.05) and accounted for all recurrence cases during follow-up.

In this study, applying traction forces of 0.5 N–2 N resulted in tissue deformation of approximately 5 mm–13 mm. Therefore, a distance of 1 cm (from the sternum notch to the innominate artery) was selected as a threshold to distinguish ETA groups, categorized as ETA (<5 mm), ETA (5–13 mm), and ETA (>13 mm), to further investigate whether these groups had differences in the number of central lymph nodes removed. The simulated venous flow velocities aligned well with the clinical data, supporting the validity of the findings. Additionally, the ETA surgery may also be affected by factors such as the surgeon’s experience and the precision of surgical instruments. We further validated our findings through follow-up studies of 288 ETA patients from two additional surgical groups (2020–2025). Notably, among patients with tumor-to-capsule distances exceeding 13 mm, seven cases demonstrated central lymph node residue and subsequent recurrence during follow-up, thereby confirming our model’s clinical feasibility (Table 2).

**TABLE 2:** Surgical characteristics and CCN recuurenc.

	Whole	low (<5mm)	Middle (5-13mm) (2)	Large (>13)(3)	*p* value	(1) versus (2)	(2) versus (3)	(1) versus (3)
Total number of CLN, mean ± SD	6.66 ± 3.21	7.39 ± 3.35	6.27±2.98	5.12 ± 2.62	0.684	0.004	0.0032	
Number of metastatic CLN,								
mean ± SD	0.74 ± 1.48	0.87 ± 1.46	0.73 ± 1.27	0.65 ± 0.88				
CCN recurrence	7	0	0	7				

While ETA remains widely used in Chinese hospitals, addressing the issue of anatomical obstructions is crucial to expanding its applicability to more patients. Although many studies report that ETA is feasible and comparable to open thyroidectomy (OT) in terms of lymph node yield and other indicators, our data indicate limitations in certain patients, particularly those with lymph nodes in Zone 7 located more than 2 cm behind the sternum. For these patients, Sun’s study suggests that ETA is not recommended, especially when lymph nodes are significantly enlarged behind the brachiocephalic artery (Sun H. et al., 2020; Sun et al., 2021).

The dissection of Zone 7 lymph nodes is of great clinical significance. Techniques such as dissection along the great vessels and the use of tracer staining (e.g., nano-carbon) can aid in better identifying thyroid lymph nodes. The thoraco-mammary approach, which operates from a foot-to-head direction, may not clearly display certain structures at the root, requiring lifting for proper identification.

For patients with distances exceeding 20 mm, ETA may still be suitable in some cases due to factors such as force application and tissue mobility. However, it is important to note that these patients have at least a 5% risk of recurrence. Alternative approaches such as conventional open thyroidectomy (COT) or transoral vestibular endoscopic thyroidectomy (TOVETA) may be more appropriate for these cases. Currently, there is a lack of quantitative factors to predict lymph node and soft tissue mobility. In this study, DD3 measurements of 21.2 mm, 23 mm, and 27 mm in three cases of central lymph node recurrence exceeded the range of soft tissue movement achievable with a maximum traction force of 2 N *in vivo*, leading to residual lymph nodes and the need for secondary surgery.

While ETA provides excellent cosmetic outcomes and high patient satisfaction with neck appearance and quality of life, there remains a blind spot in the visual field for central lymph node dissection, particularly for lymph nodes behind the sternum. Most surgeons rely on experience to judge the completeness of central lymph node dissection. In this study, we accurately predicted the movement and stretching of central lymph nodes using CT combined with numerical simulation analysis and *in vitro* stress testing, providing valuable guidance for clinical decision-making. The results of our finite element analysis were highly consistent with clinical data, showing significant differences in the number of lymph nodes removed among the 5 mm, 10 mm, and 20 mm groups. Greater distances were associated with fewer lymph nodes removed and higher rates of residual lymph nodes and recurrence during follow-up, highlighting the limitations of the endoscopic visual field. Therefore, careful preoperative evaluation is essential when selecting the ETA approach. Additionally, gender, anatomical structure, as well as the surgeon’s experience and the precisionemerged as key determinants to affect the ETA. Therefore, to ensure the accuracy of this study, we selected samples from a single surgical group with more extensive experiencee (8 years of practice) with higher accuracy surgical instruments (Storze). Specifically, male patients demonstrated lower ETA completion rates compared to female patients (the distance (>13 mm) in the male population is significantly increased, and the central area residue rate remains high after ETA surgery). However, anatomical structure and gender-related factors were not considered in our study, since our surgical approach estimation was based on anatomical data from a single subject to explore a method to evaluate the biomechanical behaviors of particular ETA patients for surgery suggestion, which warrants further investigation in future research.

Some limitations remain in the current FSI model. The innominate artery flow was assumed to be steady, ignoring the effects of respiration on flow dynamics. Additionally, the mechanical properties of soft tissue were modeled as homogeneous and isotropic, and the nonlinear viscoelastic behaviors of soft tissues were not considered. The mechanical properties of soft tissue were derived from previous studies involving healthy subjects, which cannot reflect the mechanical behavior of actual human tissues during surgery is affected by a variety of complex factors. Additionally, the mechanical properties of soft tissue are assumed as a New-hookean hyperelastic model, which omitted viscoelastic and anisotropic properties, as our study focused on short-term tissue deformation (short-term mechanical response) and modeling full anisotropy would require high-resolution, tissue-specific parameters that that are currently insufficiently characterized in the literature for our application. These simplifications may influence the accuracy of simulated tissue deformation and stress. Future work will focus on incorporating heterogeneous soft tissue properties and dynamic innominate artery flow into the FSI model to better analyze the complex biomechanical effects of traction forces on lymph nodes.

In conclusion, while ETA offers significant advantages in terms of cosmesis and patient satisfaction, its limitations in certain anatomical scenarios must be carefully considered. Improved preoperative evaluation and advanced modeling techniques can help optimize patient selection and surgical outcomes.

## 4 Conclusion

This study developed a novel FSI model to predict tissue deformation and stress in patients under traction forces, utilizing preoperative CT images to assess whether lymph nodes and soft tissues in Zone 7 could be fully cleared. The results demonstrated that patients with tissue thicknesses of <5 mm and 5–13 mm are suitable candidates for endoscopic thyroidectomy via the ETA (Table 2). Additionally, while the innominate artery flow velocities were slightly reduced under traction forces, they remained within the normal physiological range, indicating minimal hemodynamic impact during surgery.

The key contribution of this study lies in its ability to accurately evaluate preoperative feasibility for ETA, enabling surgeons to achieve the benefits of total thyroidectomy and even lateral neck lymph node dissection while maintaining a traceless neck. By combining CT imaging, FSI modeling, and clinical data, this approach provides a reliable method for patient selection, reducing the risk of incomplete lymph node clearance and recurrence. This advancement supports the broader adoption of ETA while ensuring optimal surgical outcomes and patient safety.

## Data Availability

The original contributions presented in the study are included in the article/Supplementary Material, further inquiries can be directed to the corresponding authors.
